# Low Au-content CoAu electrodes for environmental applications†

**DOI:** 10.1039/d2ra04828k

**Published:** 2022-09-15

**Authors:** Kristina Radinović, Jadranka Milikić, Aldona Balčiūnaitė, Zita Sukackienė, Marko Bošković, Loreta Tamašauskaitė-Tamašiūnaitė, Biljana Šljukić

**Affiliations:** a University of Belgrade, Faculty of Physical Chemistry Studentski trg 12-16 Belgrade 11158 Serbia; b Center for Physical Sciences and Technology Saulėtekio ave. 3 Vilnius LT-10257 Lithuania; c University of Belgrade, Institute of Chemistry, Technology, and Metallurgy, Department of Microelectronic Technologies Njegoševa 12 Belgrade 11000 Serbia; d Center of Physics and Engineering of Advanced Materials, Laboratory for Physics of Materials and Emerging Technologies, Chemical Engineering Department, Instituto Superior Técnico, Universidade de Lisboa Lisbon1049–001 Portugal biljka@ffh.bg.ac.rs

## Abstract

Six cobalt gold (CoAu) electrodes were prepared by electroless deposition using different gold-containing solutions (acidic and weakly acidic) and different Au deposition times. Characterization of CoAu electrodes was done by scanning electron microscopy with energy-dispersive X-ray spectroscopy, N_2_-sorption, and X-ray powder diffraction techniques. The possibility of using the prepared electrodes in environmental applications, *i.e.*, for the electrochemical sensing of a trace amount of arsenic(iii) in weakly alkaline media was assessed. Employing the CoAu electrode (prepared by immersing Co/Cu into 1 mM HAuCl_4_ (pH 1.8) at 30 °C for 30 s) under optimized conditions (deposition potential −0.7 V and deposition time of 60 s), a low limit of detection of 2.16 ppb was obtained. Finally, this CoAu electrode showed activity for arsenic oxidation in the presence of Cu(ii) as a model interferent as well as in real samples. Furthermore, the use of CoAu electrode as an anode in fuel cells, namely, direct borohydride – hydrogen peroxide fuel cells was also assessed. A peak power density of 191 mW cm^−2^ was attained at 25 °C for DBHPFC with CoAu anode at a current density of 201 mA cm^−2^ and cell voltage of 0.95 V, respectively. The peak power density further increased with the increase of the operating temperature to 55 °C.

## Abstract 2

A low Au-content CoAu electrode prepared by simple electroless deposition outperforms a pure Au electrode for versatile environmental applications: As(iii) sensing in water or as electrodes in direct borohydride-hydrogen peroxide fuel cells.

## Graphical abstract

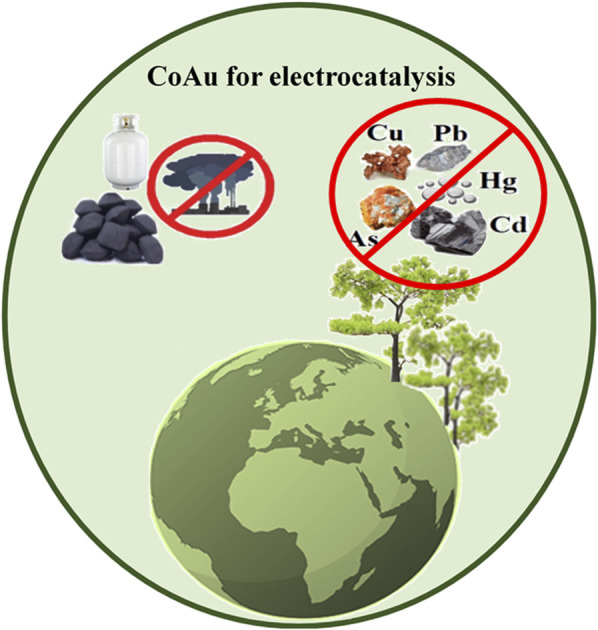

## 1. Introduction

Environmental safety and human health protection have become the primary goals in the present day. Namely, rapid economic development along with many benefits has also brought problems of environmental pollution and the depletion of fossil fuels that are currently the main energy sources. To overcome these challenging issues, research has been devoted to the development of clean and renewable energy sources along with sensors to monitor the level of environmental pollution. Among different types of novel energy technologies and sensors, electrochemical energy conversion devices and electrochemical sensors have been pointed out as especially promising.

To operate with high efficiency and selectivity, these devices typically need to employ highly active electrocatalysts. Gold (Au)-based materials are seen as promising for various electrocatalytic reactions due to their high catalytic activity and selectivity.^1^ Au-based nanomaterials can be prepared by different methods that will further determine their composition, morphology, and particle size, and consequently, their electrochemical response. Within the present paper, we report a simple and fast electroless deposition procedure for the preparation of CoAu electrodes and we next focus on the use of the prepared CoAu electrodes for electrochemical sensing (with arsenic(iii) as model analyte) and for electrocatalytic applications in fuel cells (namely, for borohydride oxidation reaction (BOR)). Au metal has demonstrated high activity for the electrochemical sensing of trace amounts of As(iii),^2^ as well as high activity for BOR along with low activity for parasitic borohydride (BH_4_^−^) hydrolysis reaction (more details below).^3^ Non-noble metals such as Co have been investigated for instance, for BOR;^4^ however, they typically show more pronounced activity for a hydrolysis reaction.^5^ Thus, we aim to keeping the high efficiency of Au-based electrodes but notably lowering the price of the electrode material.

Arsenic (As), a model analyte tested herein, is undoubtedly one the most toxic ions in the group of heavy metal ions^6,7^ and it exists both naturally and in industrial effluents.^8–12^ Dominant forms of inorganic arsenic species in natural water are As(iii) (AsO_3_^3−^) and As(v) (AsO_4_^3−^)^8–12^ with the former being about 60–70 times more toxic than As(v). The World Health Organization has limited the maximum concentration of arsenic in natural water to as low as 10 ppb.^6,12–14^ Therefore, fast and accurate detection of As(iii) in the environment is particularly significant. Various analytical methods, such as atomic flame absorption spectrophotometry, hydride-generation atomic absorption, emission spectrometry, inductively coupled plasma optical emission spectrometry, and inductively coupled plasma mass spectrometry have been traditionally used for the detection of heavy metals.^7^ However, these instrumental methods require specialised laboratory conditions and lengthy sample preparation. On the other hand, electrochemical methods are highly selective, and fast and require cheaper and portable equipment suitable for field analysis.^15,16^ Different types of Au-based electrodes were tested as arsenic sensors in aqueous media. Three-dimensional porous graphitic carbon nitride decorated by Au nanoparticles (AuNPs/g-C_3_N_4_) gave a response in the presence of As(iii) species in different real samples, including tap water, spring water from the mountain, and the water from the river.^2^ Alloys of Au and Rare Earth (RE) elements showed good performance for the electroanalytical determination of As(iii) in weakly alkaline media. The limit of detection (LOD) of As(iii) obtained with the tested alloys was far below the WHO value and increased in the order: Au–Ho (0.8 ppb) < Au–Dy (1.5 ppb) < Au–Y (1.6 ppb) < Au–Sm (2.3 ppb).^6^ Gold–copper (Au–Cu) bimetallic nanoparticles prepared by the hydrothermal method presented high performance as arsenic sensors with high sensitivity at ppb level where Au_89_Cu_11_ electrodes with the highest content of Cu showed the best stripping behaviour and the highest sensitivity for As(iii) detection.^17^ Electrodes based on cobalt oxide nanoparticles (CoO_*x*_) showed to be excellent arsenic sensors at a wide pH range from 5 to 11^18^ displaying a clear peak corresponding to arsenic oxidation.

As mentioned, another important aspect of preserving environmental safety is the reduction of greenhouse gases emission from the combustion of fossil fuels. Thus, energy needs to be generated using clean energy sources such as fuel cells. The direct borohydride fuel cells, which use an aqueous solution of borohydride (NaBH_4_) as a fuel and oxygen (DBFCs) or hydrogen peroxide (DBHPFCs) as the oxidant, are considered to have great energy potential and high energy density.^19^ The reaction taking place at the DBFC anode, BOR, can involve a maximum of eight electrons, according to eqn (1):.^20,21^

1 BH_4_^−^ + 8OH^−^ → BO_2_^−^ + 6H_2_O + 8e^−^ *E*^0^ = 1.24 V *vs.* SHE

It is known that in practice it is difficult to achieve the transfer of eight electrons due to the parallel hydrolysis of BH_4_^−^ (eqn (2)), a spontaneous reaction that consumes part of the BH_4_^−^ ions present in the solution and generates hydrogen.^22^

2 BH_4_^−^ + 2H_2_O → BH_2_^−^ + 4H_2_

Au typically has a lower activity for hydrolysis reaction,^23^ so it can lead to the indicated number of exchanged electrons.^24–26^ The disadvantage is the slow BOR kinetics on the Au anode, which leads to low power output and poor electrochemical performance of DBFCs.^27^ Studies have shown that alloying Au with lower-cost metals can improve catalytic performance and, at the same time, reduce the cost of the material. Promising results have been obtained for a variety of non-noble metals, such as Co, Ni, Cu, Zn, and Fe.^28^ Pei *et al.* reported that supported Au–Co alloy catalysts have higher catalytic activity for the direct oxidation of BH_4_^−^ than pure nano-sized Au catalyst, especially the Au_45_Co_55_/C catalyst.^29^ Catalytic activity for BOR of a series of electrocatalysts based on Au modified with Zn was reported to be higher than in the case of a pure Au electrode.^30^ Au–RE: Au–Sm, Au–Dy, Au–Ho, and Au–Y, alloys tested for BOR showed significantly higher current densities compared to the Au electrode for BOR.^28^

Thus, as mentioned, we herein present an easy synthesis of CoAu electrodes by simple electroless deposition and characterise them using scanning electron microscopy/energy dispersive X-ray spectroscopy (SEM-EDS), N_2_-sorption and X-ray diffraction (XRD) techniques. The anodic stripping voltammetry (ASV) method was used for As(iii) detection while cyclic voltammetry and chronoamperometry were used for the BOR studies.

## 2. Experimental

### 2.1. Reagents and materials

A stock solution of As(iii) (1 mM) was prepared by dissolving 3.25 mg of sodium arsenite (NaAsO_2_, Fisher Chemical) in 25 cm^3^ of deionized water (Simplicity® UV Water Purification System, Merck Millipore). Sodium hydrogen carbonate (NaHCO_3,_ Zdravlje Leskovac) and sodium carbonate anhydride (Na_2_CO_3_, Zorka Šabac) were used for the preparation of NaHCO_3_ + Na_2_CO_3_ buffer. Copper sulfate (CuSO_2_, NRK Engineering Belgrade) was used for studying the influence of interferents on the electrode's response to As(iii). Sodium borohydride (NaBH_4_, Scharlau, 97 wt%) and sodium hydroxide (NaOH, Sigma-Aldrich) were used for the preparation of anolyte, while H_2_O_2_ (30 vol%, Carlo Erba) and HCl (37 wt%, Sigma Aldrich) were used for the preparation of catholyte. All solutions were prepared using deionized water (Simplicity® UV Water Purification System, Merck Millipore).

### 2.2. Preparation of gold cobalt electrodes

Electroless deposition of Co was performed on the copper surface with a prior activation step with Pd(ii) ions. Briefly, prior to electroless deposition of Co, Cu sheets (1 cm × 1 cm) were pre-treated with 50–100% calcium magnesium oxide, known as “Vienna Lime” (Kremer Pigments GmbH & Co. KG), and rinsed with deionized water. Then the Cu sheets were placed into the electroless cobalt bath containing 0.05 M CoSO_4_, 0.05 M C_4_H_8_ONH. BH_3_ and 0.2 M glycine (NH_2_CH_2_COOH). The bath operated at pH 7 at a temperature of 30 °C for 30 min. The temperature of 30 °C was observed to be the optimum one for the deposition of these coatings. At a lower temperature, the coatings were applied unevenly and not smoothly. At a higher temperature, the adhesion became worse and the coatings started to “crumble” and to have some cracks. The thickness of the pure Co coatings determined gravimetrically was *ca.* 1 μm.^31,32^ Transmission electron microscopy (TEM) analysis of the deposited Co coating was carried out using FEI TECNAI F20 field emission microscope operating at 200 kV.

Au nanoparticles were deposited on the prepared Co/Cu electrodes by galvanic displacement of Au at a temperature of 30 °C from a 1 mM HAuCl_4_ solution (pH 1.8) (denoted as an acidic Au-containing solution) or from a 1 g l^−1^ KAu(CN)_2_ + 0.4 M (NH_4_)_2_C_6_H_6_O_7_ complex (pH 5) (denoted as a weak acid Au-containing solution).

The immersion periods of the Co/Cu electrodes into the gold-containing solutions were 0.5, 1, and 5 min. After plating, the samples were taken out, thoroughly rinsed with deionized water, and dried in air at room temperature.

### 2.3. Characterization of CoAu electrodes

XRD analysis (with X-ray diffractometer D2 Phaser Bruker AXS) and SEM-EDX (with scanning electron microscope TM4000Plus Hitachi) were conducted to provide comprehensive information about the structure and dispersion of active components on the surface of the electrodes prepared. XRD patterns were recorded using Cu Kα radiation. A step-scan mode was used in the 2*θ* range from 30° to 90° with a step length of 0.04° and a counting time of 1 s per step. Sorption isotherm of CoAu (30 s, pH 1.8) was obtained by nitrogen sorption at 77 K using a Quantachrome Instruments NOVA touch 2LX device. Prior to adsorption, the sample was degassed for 15 min at 80.0 °C, then 30 min at 120.0 °C, and finally for 180 min at 300.0 °C. The specific surface area of the sample, *S*_BET_, was calculated using the multi-point Brunauer–Emmett–Teller (BET) method.

### 2.4. DFT calculation studies

PWscf code^33^ of Quantum espresso program package was used for all the spin-polarized DFT calculations, within generalized gradient approximation (GGA) using the PBE^34^ functional formulation. Projector augmented wave (PAW) pseudopotentials were employed to describe the interactions between ionic cores and valence electrons.^35^ The valence electronic states were expanded in plane-wave basis sets with a cutoff energy of 35 Ry (476 eV). The adsorption energy was calculated as *E*_ads_ = *E*_mol/surf_ − (*E*_surf_ + *E*_mol_), where *E*_mol_, *E*_surf_, and *E*_mol/surf_ are the total energy of H_3_AsO_3_ molecules/Arsenic atoms, and gold/cobalt layer without and with H_3_AsO_3_ molecules/Arsenic atoms. A larger negative *E*_ads_ value means a more stable configuration.

### 2.5. Electrochemical measurements

#### 2.5.1 Arsenic(iii) sensing

The As(iii) electroanalytical sensing investigations were performed using PalmSense EmStat3 Blue Potentiostat within a glass cell of 25 ml volume with a standard three-electrode system. A carbon graphite rod and saturated calomel electrode (SCE) were employed as counter and reference electrodes, respectively. Thus, all potentials in this work are given *vs.* SCE reference. NaHCO_3_ + Na_2_CO_3_ buffer (pH 9.2–10.6) was used as supporting electrolytes for all electrochemical measurements related to As(III) detection. Alkaline media was used due to the possible instability of CoAu electrodes in acidic media. All As(iii) sensing studies were conducted at room temperature under a nitrogen (N_2_, Messer) atmosphere.

The electrochemical characterization of CoAu electrode was performed by cyclic voltammetry (CV) in NaHCO_3_ + Na_2_CO_3_ buffer (pH in the 9.2–10.6 range). An area of 1 cm^2^ of CoAu working electrode was exposed to the electrolyte and used for calculating current densities.

Electroanalysis of As(iii) presence was investigated by anodic stripping voltammetry (ASV)^31^ with optimization of the working parameters: scan rate, deposition potential, *E*_d_, and deposition time, *t*_d_. The impact of different *E*_d_ (−0.3 to −0.9 V) and *t*_d_ (30 to 360 s) on As(iii) oxidation was examined.

For comparison purposes, a pure Au electrode with a geometric area of 0.19625 cm^2^, was also tested.

The real water samples from the river Danube and river Begej were diluted with NaHCO_3_ + Na_2_CO_3_ buffer (sample: buffer 75 : 25 vol% ratio).

#### 2.5.2 Borohydride oxidation reaction study

Initial studies of Co and CoAu electrodes' activity for BOR were carried out by cyclic voltammetry and chronoamperometry in 0.05 M NaBH_4_ + 1 M NaOH at 25 °C. DBHPFC tests were carried out by employing the Co and CoAu electrodes with a geometric area of 2 cm^2^ as the anode and a Pt sheet as the cathode. The catholyte was composed of an alkaline mixture of 1 M NaBH_4_ + 4 M NaOH and the anolyte contained 5 M H_2_O_2_ + 1.5 M HCl. Each compartment of the cell contained 100 ml of the corresponding aqueous electrolyte. In order to prevent H_2_O_2_ decomposition and possible loss of BH_4_^−^ by hydrolysis during storage, the test solutions were prepared immediately before the measurements. A Nafion N117 membrane was used to separate the anodic and cathodic compartments of the single direct NaBH_4_/H_2_O_2_ fuel cell. The presented current densities are normalized with respect to the geometric area of electrodes. All electrochemical measurements were performed with a Zennium electrochemical workstation (ZAHNER-Elektrik GmbH & Co. KG). The performance of the fuel cell was evaluated by recording the cell polarization curves and constructing the corresponding power density curves.

## 3. Results and discussion

### 3.1. Characterization

**Scheme 1:**
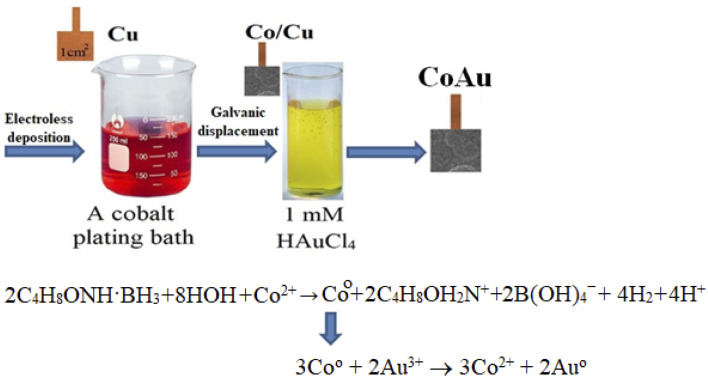
Schematic representation of material synthesis by electroless deposition.

The initial stage of Co deposition on the substrate, anodic oxidation of morpholine borane (C_4_H_8_ONH·BH_3_, MB), can be presented as follows (eqn (3)) (Scheme 1):

3 C_4_H_8_ONH·BH_3_ + 3HOH + OH^−^ → C_4_H_8_OH_2_N^+^ + B(OH)_4_^−^ + 5H^+^ + 6e^−^

The electrons released within this reaction are consumed for the reduction of cobalt(ii) ions (eqn (4)) and boron (eqn (5)):

4 2C_4_H_8_ONH·BH_3_ + 8HOH + Co^2+^ → Co + 2C_4_H_8_OH_2_N^+^ + 2B(OH)_4_^−^ + 4H_2_ + 4H^+^

5 2C_4_H_8_ONH·BH_3_ + 2H^+^ → 2BH_3_ + 2C_4_H_8_OH_2_N^+^ → 2B + 2C_4_H_8_OH_2_N^+^ + 3H_2_

At the same time, some quantity of morpholine borane may be additionally consumed in the following reaction (eqn (6)):

6 C_4_H_8_ONH·BH_3_ + 4H_2_O → C_4_H_8_OH_2_N^+^ + B(OH)_4_ + 3H_2_

Fig. S1† shows TEM image of the cross section of Co deposited onto Cu under conditions described in the Experimental section. The rate of Co deposition and the composition of the Co coatings were observed to depend on the solution pH and MB concentration.^36^ Increase of pH (from 6 to 8) accelerates the rate of Co reduction most likely due to the acceleration action of OH^−^ ions to the first step of the process – morpholine borane anodic oxidation (eqn (3)). Furthermore, increase of pH led to a lower amount of boron incorporated in Co coatings.

With the increase in morpholine borane concentration in the solution, its anodic oxidation accelerated and, consequently, the Co deposition rate increased markedly as well. This rise in Co deposition rate with increase in concentration of MB in the solution was also reflected in the changes in the composition of the coatings, *i.e.*, in the decrease of the quantity of Co in coatings.

The immersion deposition of Au particles is a galvanic replacement reaction. Namely, due to the difference between standard potentials of the Co^2+^/Co and Au^3+^/Au pairs (eqn (7) and (8), respectively), the reaction (eqn (9)) takes place:

7 2Au^3+^ + 6e^−^ → 2Au *E*_o_ = 1.68 V (SHE)

8 3Co → 3Co^2+^ + 6e^−^ *E*_o_ = −0.28 V (SHE)

9 3Co^0^ + 2Au^3+^ → 3Co^2+^ + 2Au^0^

**Fig. 1:**
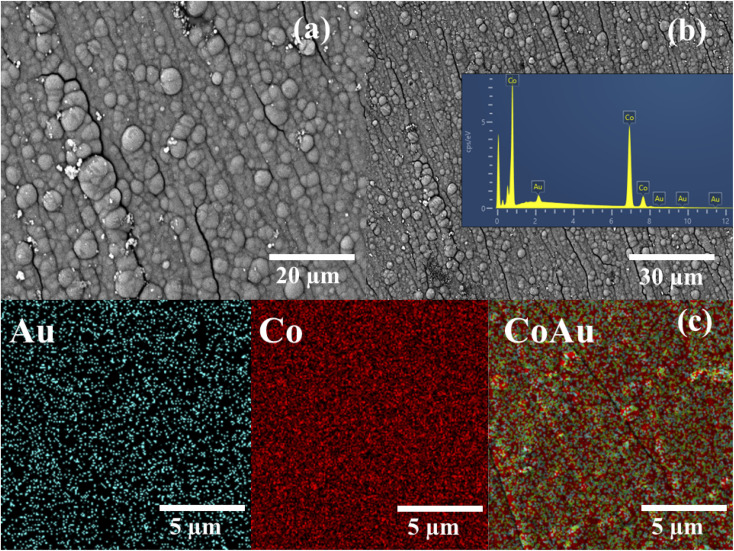
(a and b) SEM images of CoAu (30 s, pH 1.8) at different magnifications with EDS spectrum (inset) and (c) SEM-EDS mapping of CoAu (30 s, pH 1.8) electrode.

Fig. 1a and b presents SEM images of CoAu (30 s, pH 1.8) electrode at different magnifications where Au nanoparticles visible as bright crystallites of mostly round shape are evenly distributed over the entire surface of the Co-coated electrode. SEM-EDS mapping of the surface of CoAu (30 s, pH 1.8) electrode (Fig. 1c) shows the element distribution where Co and Au nanoparticles are homogeneously dispersed on the copper surface.

Amounts of metals deposited on the CoAu (30 s, pH 1.8) electrode surface were determined by EDS and found to be 96.41 and 3.59 wt% for Co and Au, respectively (Table 1).

**Table 1:** Surface weight (%) and atomic composition (%) of the CoAu (30 s, pH 1.8) electrode determined by EDS analysis

Whole surface		
	Weight (%)	Atomic (%)
Co	96.41	98.90
Au	3.59	1.10

Specific surface area of CoAu (30 s, pH 1.8) according to the BET isotherm, *S*_BET_, was found to be 30 m^2^ g^−1^.

**Fig. 2:**
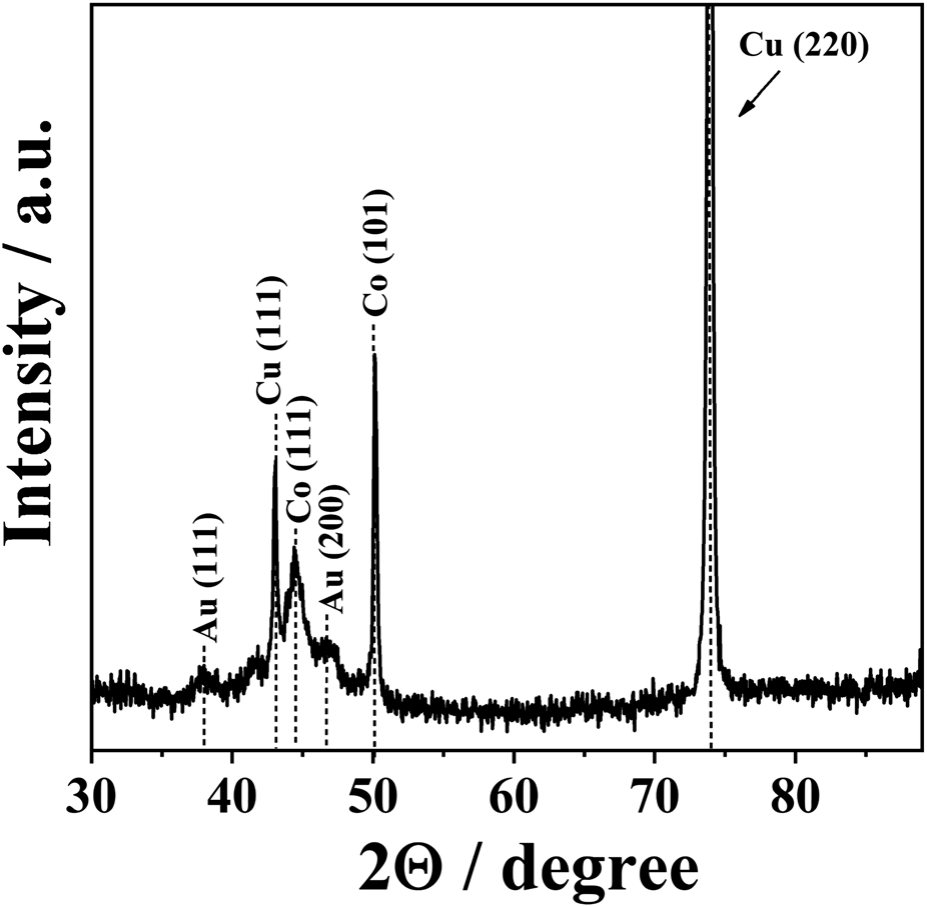
XRD pattern of CoAu (30 s, pH 1.8) electrode.

Additional structural characterization of CoAu (30 s, pH 1.8) electrode was done by XRD analysis (Fig. 2). The reflections from Co(111) and Co(101) planes were observed at 2*θ* of 44.4 and 50.1°, respectively.^37^ The low-intensity peaks at 2*θ* of 37.9 and 46.5° originate from the reflections from Au(111) and Au(200) crystalline planes, respectively.^3^ The highest intensity peak at 2*θ* of 73.9° and a lower intensity peak at 2*θ* of 43.1° correspond to the reflection from Cu(220) and Cu(111) planes of Cu substrate, respectively.^38,39^

**Fig. 3:**
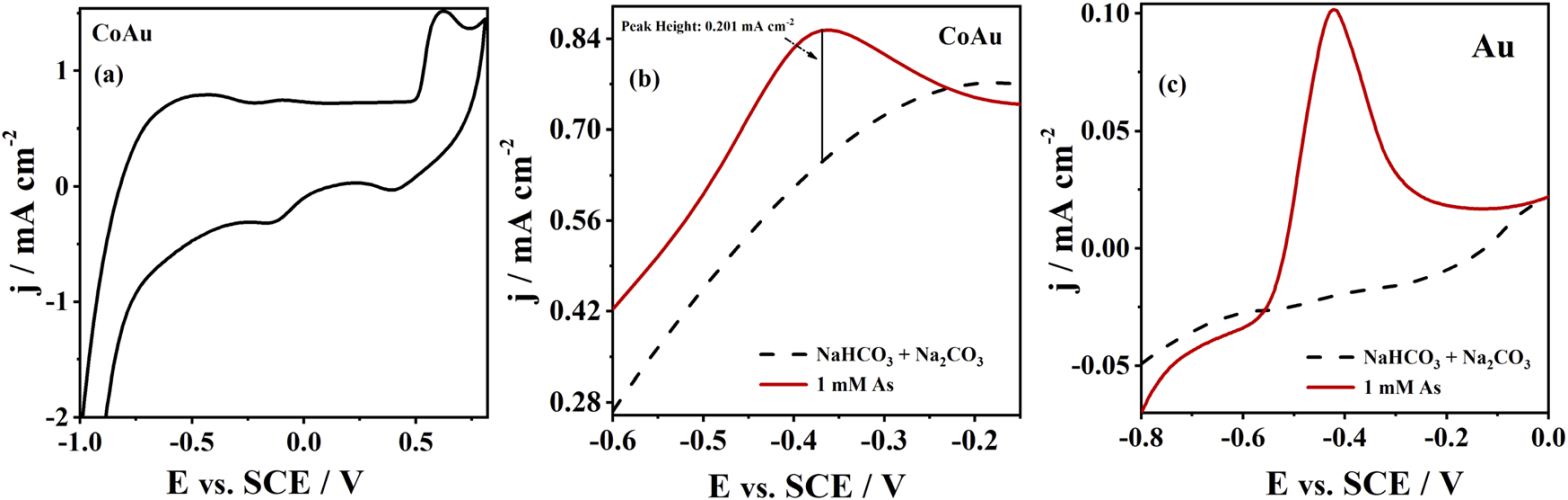
(a) CV of CoAu electrode in NaHCO_3_ + Na_2_CO_3_ buffer, ASV of (b) CoAu electrode, and (c) bulk Au electrode in NaHCO_3_ + Na_2_CO_3_ buffer in the absence and in the presence of As(iii) (1 mM) recorded at a scan rate of 50 mV s^−1^ after holding the potential at −0.7 V for 60 s.

The electrochemical characterization of CoAu (30 s, pH 1.8) electrode in NaHCO_3_ + Na_2_CO_3_ buffer (Fig. 3a) shows the oxidation of Au at *ca.* 0.6 V with a corresponding reduction peak at *ca.* 0.4 V on the reverse scan.^40^

### 3.2. As(iii) sensing using CoAu electrode

Two sets of CoAu electrodes were tested for electroanalytical detection of As(iii) in a weakly alkaline medium (NaHCO_3_ + Na_2_CO_3_ buffer). The first set of electrodes was prepared by immersing Co/Cu into 1 mM HAuCl_4_ (pH 1.8) at 30 °C for different periods (30 s, 60 s, and 300 s) and all three prepared electrodes were active for detection of As(iii). The activity for As(iii) sensing was evidenced by the appearance of an oxidation peak in the voltammograms; Fig. S2a† illustrates the case of electrode prepared by immersion during 30 s. However, the electrodes prepared with 60 s and 300 s became passive after some time as evidenced by the disappearance of the previously observed oxidation peak, Fig. S2b and c.† The second set of electrodes was prepared by immersing Co/Cu into 1 g l^−1^ KAu(CN)_2_ + 0.4 M (NH_4_)_2_C_6_H_6_O_7_ complex (pH = 5) at 30 °C for various periods (30 s, 60 s, and 300 s) and all prepared electrodes were inactive for As(iii) detection. Namely, no peak corresponding to As oxidation was observed when using such fabricated CoAu electrodes, Fig. S2d, e and f.† Therefore, the CoAu (30 s, pH 1.8) electrode prepared by immersing Co/Cu into 1 mM HAuCl_4_ (pH 1.8) for 30 s was examined in detail (and it is from this point onward refereed in the text just as CoAu electrode).

Voltammogram of CoAu electrode in the presence of As(iii) showed a clear peak of As(0) oxidation to As(iii), Fig. 3b. As(0) oxidation at the potential of *ca.* −0.36 V was also reported when testing As(iii) sensing using Au–RE (RE = Sm, Ho, Dy, Y) electrodes in the same buffer.^6^ The oxidation peak reached a current density of 0.20 mA cm^−2^ (current density difference in the presence and in the absence of As(iii) at −0.36 V). Fig. 3c illustrates the behaviour of a bulk Au electrode that gave *ca.* 8.5 times lower peak current density in the presence of As(iii) relative to the CoAu electrode.^41^

**Scheme 2:**
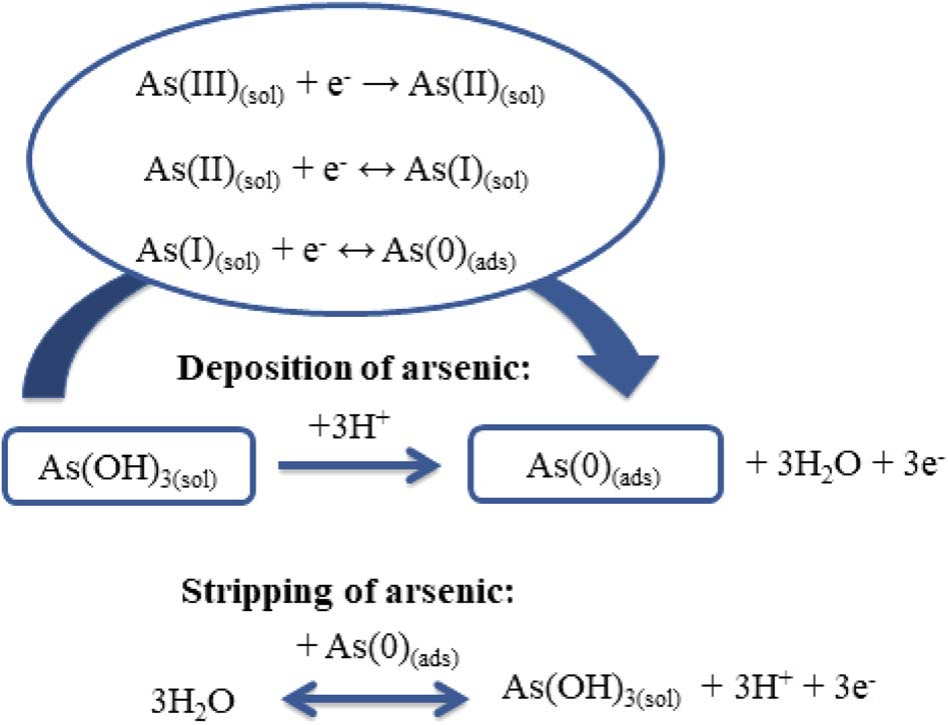
Schematic representation of the electroanalytical sensing of As(iii).

It is known that the determination of As(iii) using ASV involves two steps:^42^ during the first step, As(iii) is reduced to As(0) and adsorbed on the electrode surface (eqn (10)); then the adsorbed As (0) is removed from the electrode surface back to the solution by oxidation to As(iii) (eqn (11)) (Scheme 2).^7,18,35^

10 Deposition: As^3+^ + 3e^−^ → As^0^

11 Stripping: As^0^ → As^3+^ + 3e^−^

Theoretical considerations of the mechanism of As(iii) deposition (eqn (12)–(14)) have indicated transfer of the first electron as the rate-determining step:^7^

12 As(iii)_(sol)_ + e− → As(ii)_(sol)_

13 As(ii)_(sol)_ + e^−^ ↔ As(i)_(sol)_

14 As(i)_(sol)_ + e^−^ ↔ As(0)_(ads)_

In order to gain insight into the adsorption process, the adsorption energy of arsenic acid and atomic arsenic on the planes of gold(111) and (200) and on the planes of cobalt(111) and (101) were calculated, Table 2. The adsorption energies of arsenic acid correspond to chemisorption, while the energy in the case of arsenic atoms corresponds to the formation of a metal layer on the surfaces.

**Table 2:** Results of calculation of the adsorption energy of H_3_AsO_3_ and As atomic

	*E*_ads_ (H_3_AsO_3_) [kJ mol^−1^]	*E*_ads_(As) [kJ mol^−1^]
Au(111)	−190	−1259
Au(200)	−147	−1703
Co(111)	−122	−760
Co(101)	−234	−988

**Fig. 4:**
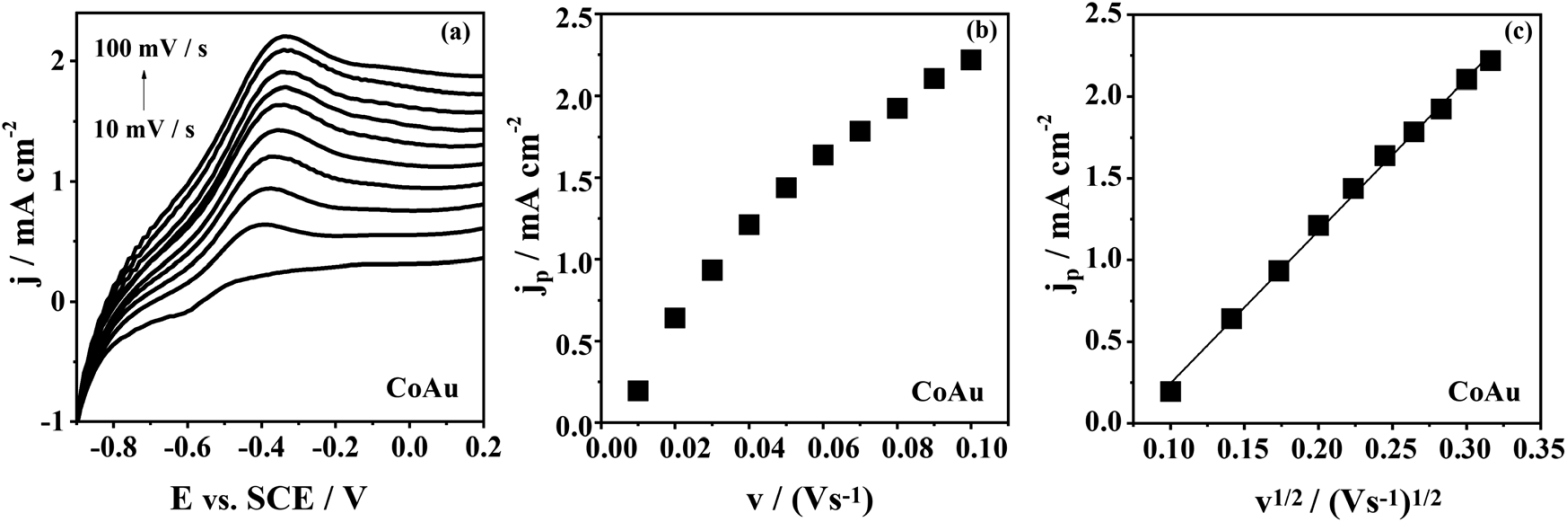
(a) Voltammograms of CoAu electrode at different scan rates after holding the potential at −0.7 V for 60 s with, (b) the corresponding *j*_p_*vs. ν* plot of CoAu electrode, and (c) the corresponding *j*_p_*vs. ν*^1/2^ plot of CoAu electrodes.

The effect of the scan rate on the arsenic oxidation at CoAu electrode in NaHCO_3_ + Na_2_CO_3_ buffer (Fig. 4) is demonstrated as shifting of the oxidation peak to more positive potential values with increasing the scan rate. This behaviour of CoAu electrode during As(0) to As(iii) oxidation is typical for an irreversible process.^43^Fig. 4(b and c) shows the peak current density corresponding to oxidation of As(0) to As(iii) as a function of scan rate and the square root of scan rate, respectively. The coefficient of determination (*R*^2^) of the *j*_p_*vs. ν*^1/2^ plot is found to be 0.99975 indicating that diffusion of active species plays (Scheme 1) role in arsenic oxidation.^44^

**Fig. 5:**
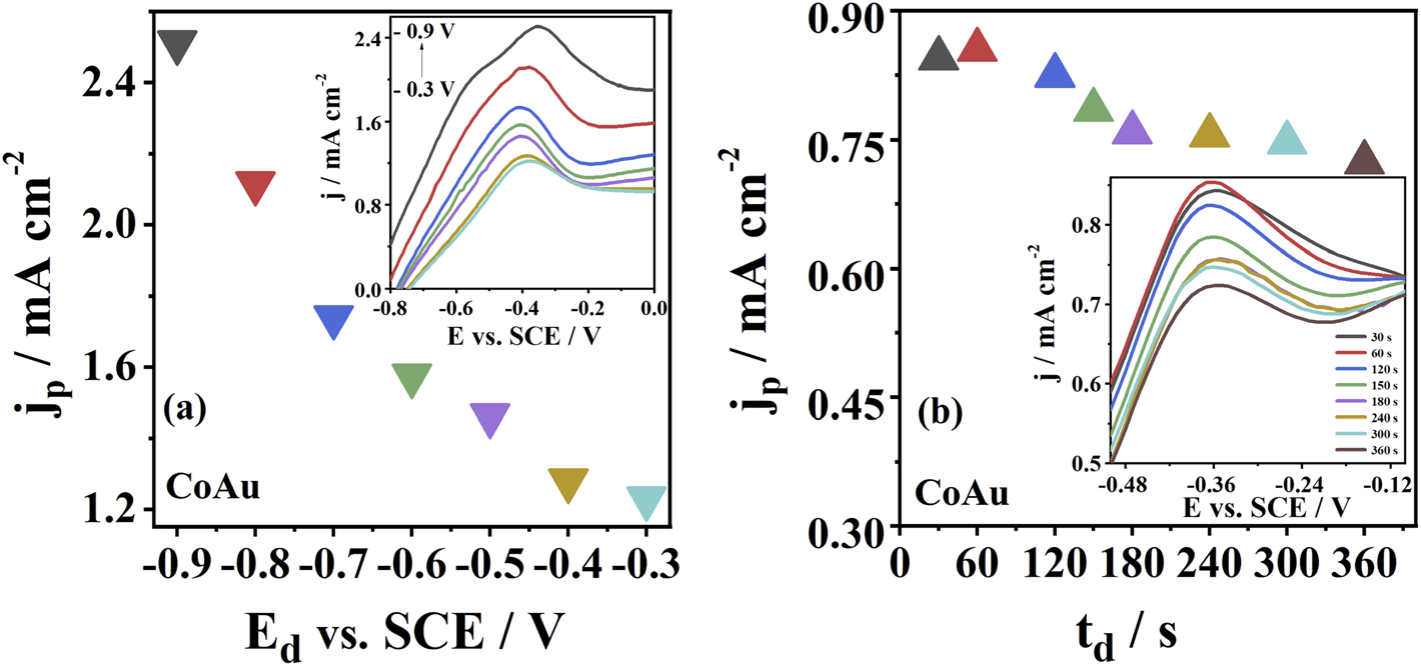
(a) Voltammograms of CoAu electrode recorded using different deposition potentials, *E*_d_, during 180 s with the corresponding *j*_p_*vs. E*_d_ plot (inset). (b) Voltammograms of CoAu electrode recorded using different deposition times, *t*_d_, (at −0.7 V) with the corresponding *j*_p_*vs. t*_d_ graph (inset). All measurements were done in 1 mM As(iii) in NaHCO_3_ + Na_2_CO_3_ buffer at a scan rate of 50 mV s^−1^.

Furthermore, the deposition process, *i.e.*, deposition parameters (*E*_d_ and *t*_d_), were optimised. The highest As(iii) oxidation peak current density at CoAu electrode was obtained for the deposition potential of −0.9 V, decreasing at less negative potentials (Fig. 5a). Still, a deposition potential of −0.7 V was selected for further measurements to avoid subjecting the electrode to high negative potentials. The highest current density of 0.85 mA cm^−2^ of CoAu electrode was obtained at a deposition time of 60 s (Fig. 5c). Namely, the current density for a deposition time of 30 s was slightly lower (0.84 mA cm^−2^) than for 60 s (0.85 mA cm^−2^). Unexpectedly, the peak current density of CoAu electrode was decreased above 60 s where the lowest current density of 0.72 mA cm^−2^ was obtained for 360 s (Fig. 5d).

**Fig. 6:**
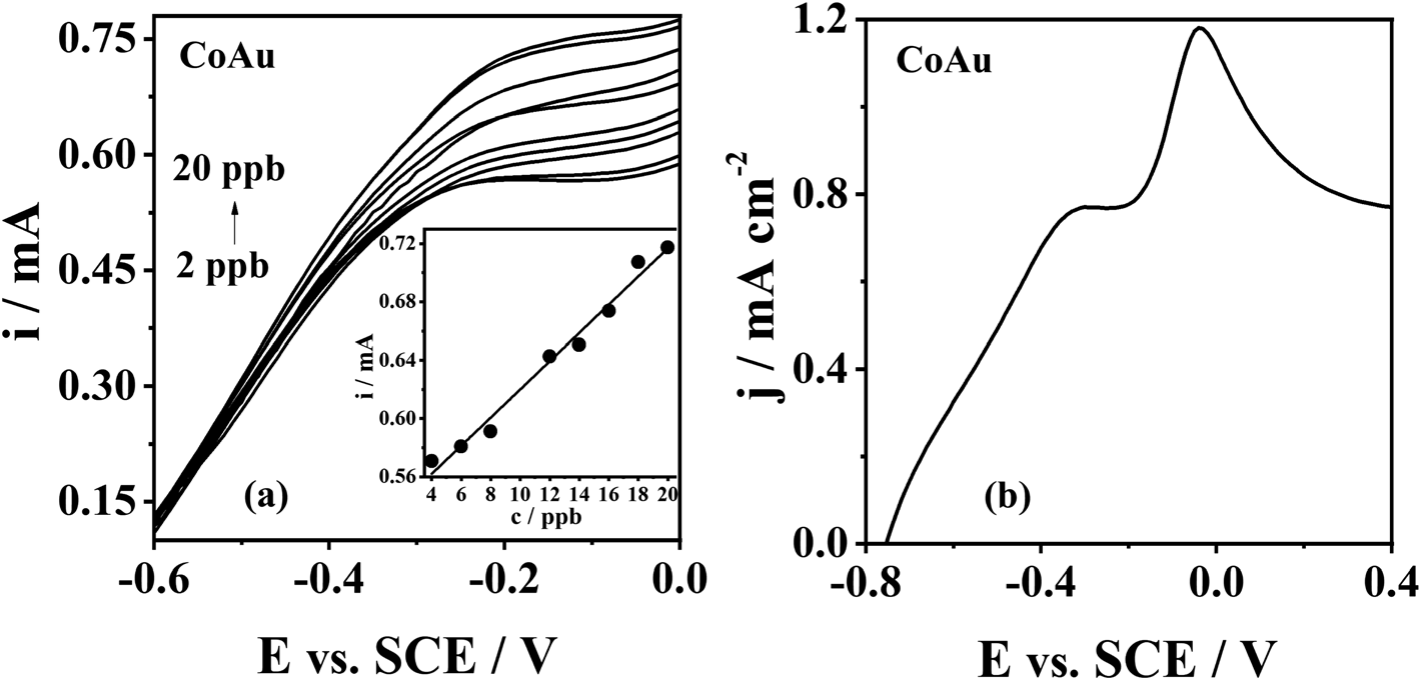
(a) Voltammograms of CoAu electrode in NaHCO_3_ + Na_2_CO_3_ buffer with increasing concentrations of As(iii) (2 to 20 ppb) with the corresponding standard addition plots (inset). (b) Voltammogram of CoAu electrode in As (1 mM) + Cu (1 mM) solution in NaHCO_3_ + Na_2_CO_3_ buffer. Voltammograms recorded at a scan rate of 50 mV s^−1^ after holding the potential at −0.7 V for 60 s.

The standard addition plot of As(iii) sensing using CoAu electrode was obtained under the optimised deposition conditions. The increase of peak current density with the increase of the As(iii) concentration in the range from 2 to 20 ppb could be seen (Fig. 6a) and the corresponding peak current, *i*_p_, *vs.* concentration, *c*, plot data (Fig. 6a(inset)) were used for determining the LOD value using the 3 sigma method.^45^ Thus, LOD of As(iii) sensing with CoAu electrode was found to be as low as 2.16 ppb. The obtained value is well below the maximum allowed As concentration (10 ppb) in drinking water set by WHO^46^ suggesting that the herein-tested CoAu electrode could be a good electrode material for As(iii) sensing. Further, this value is lower than the LOD of 25.98 ppb obtained with the glassy carbon electrode (GCE) modified with nano Au-crystal violet film in pH 7 PBS (linear range 259.82–2598.2 ppb) by differential pulse voltammetry under the optimized conditions.^47^ Also, the obtained LOD value for CoAu electrode is lower than the LOD value for Au–Sm alloy (2.3 ppb) tested in the same buffer at pH 9.2 by the ASV.^6^ On the other hand, a lower LOD of 0.13 ppb was achieved employing a carbon paste electrode modified with Au NPs – reduced graphene oxide composite in a 0.1 M phosphate buffer (pH 7.2).^48^ Square-wave ASV response of nanocomposite of α-MnO_2_ with Au NPs led to a low LOD of 0.019 ppb at pH 9.0 Na_2_CO_3_–NaHCO_3_ buffer solution (0.1 M), while at pH 5 a higher LOD was obtained for the same electrode.^49^ MnO_*x*_ with Au NPs fabricated for the detection As(iii) in alkaline media (0.1 M Na_2_CO_3_–NaHCO_3_, pH 10.0) gave a low LOD of 0.057 ppb for As(iii) sensing.^50^

Real samples, such as river water and tap water, can contain various ions including Na(i), K(i), Mg(ii), Cu(ii), Sb(iii), NO_3_^−^, F^−^, Cl^−^, with Cu(ii) being the main interfering metal ions during arsenic detection.^51^ The peak of Cu(ii) oxidation appears at a slightly more positive potential compared to the peak of As(iii) oxidation.^51^ Therefore, CoAu electrode response to As(iii) (1 mM) was examined in presence of Cu(ii) as model interferente. The voltammogram of the herein examined electrode showed the presence of two oxidation peaks (Fig. 6b) indicating that As(iii) response is not affected by the presence of Cu(ii) cation in NaHCO_3_ + Na_2_CO_3_ buffer. The first peak at *ca.* −0.3 V corresponds to As oxidation and the second peak at *ca.* −0.04 V is related to Cu oxidation.^52^

**Fig. 7:**
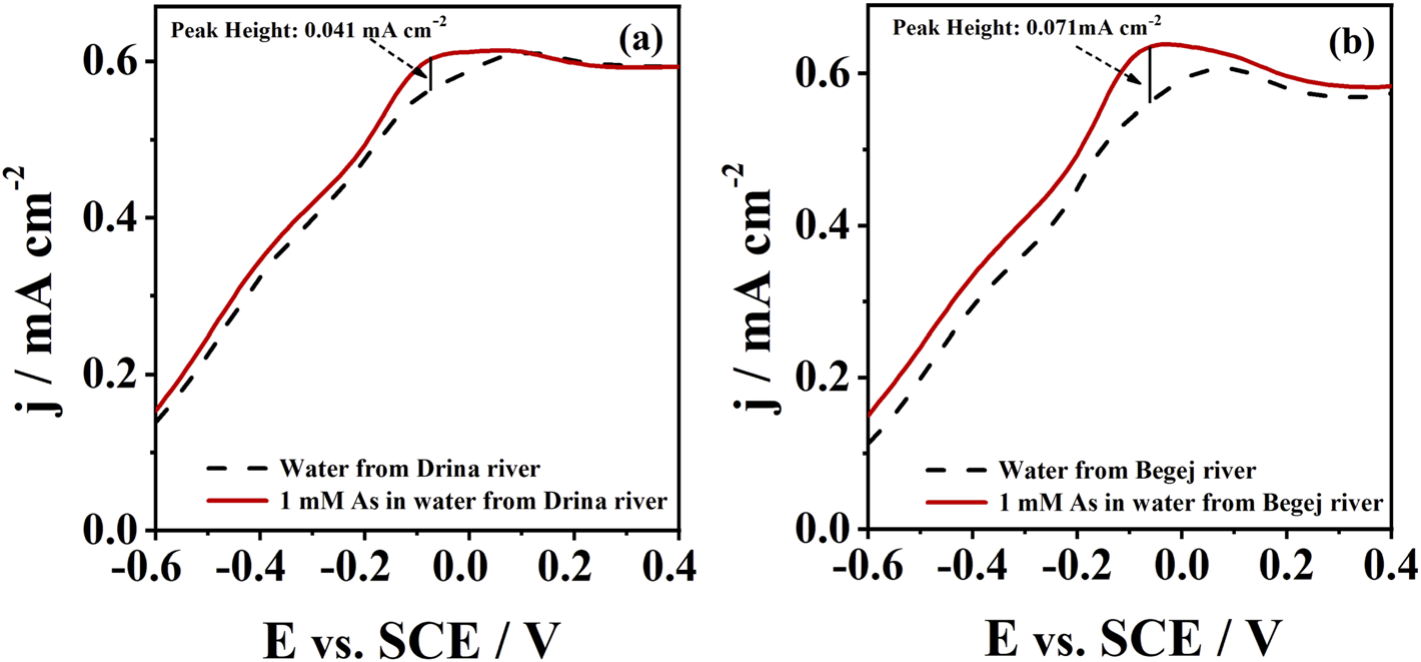
Voltammograms of CoAu electrode in (a) river Drina and (b) river Begej in the absence (- - -) and in the presence (—) of As(iii) (1 mM) recorded at a scan rate of 50 mV s^−1^ after holding the potential at −0.7 V for 60 s.

The potential application of CoAu electrode for arsenic detection in a real water sample was assessed and CoAu electrode showed activity for As(iii) sensing in two river samples, Fig. 7. The peak corresponding to As(0) oxidation was observed in the diluted samples (without any pretreatment) at *ca.* −0.1 V. Peak current of river Drina sample reached 0.041 mA cm^−2^ while peak current of river Begej sample reached 0.071 mA cm^−2^.

### 3.3. Borohydride oxidation reaction studies

**Fig. 8:**
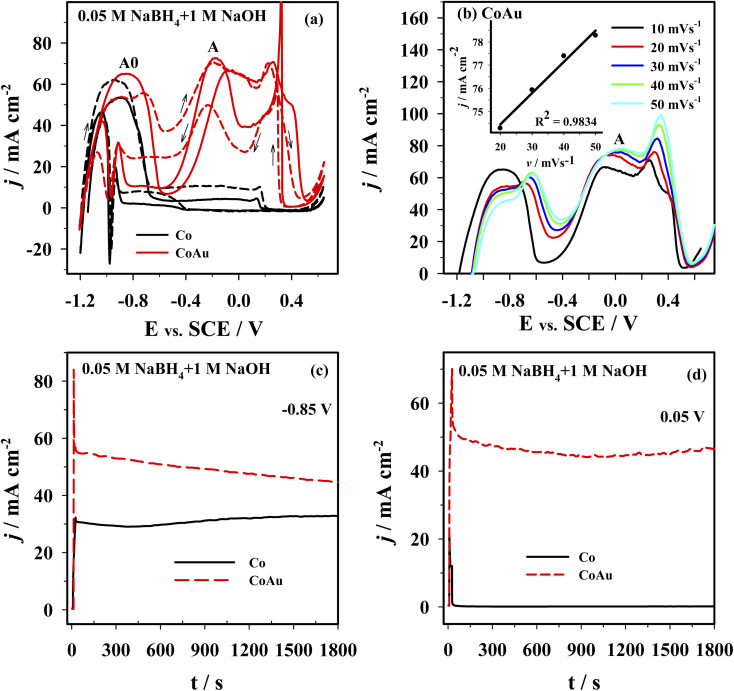
(a) CVs of Co (black line) and CoAu (red line) electrodes at a scan rate of 10 mV s^−1^: 1st cycle (solid line) and the 5th cycle (dashed line). (b) Positive-going potential scans for the Co and CoAu electrodes at different scan rates. (c and d) Chronoamperometric data of Co and AuCo electrodes at a potential of −0.85 V (*vs.* SCE) and 0.05 V (*vs.* SCE). All measurements were done in 0.05 M NaBH_4_ + 1 M NaOH at 25 °C.

**Scheme 3:**
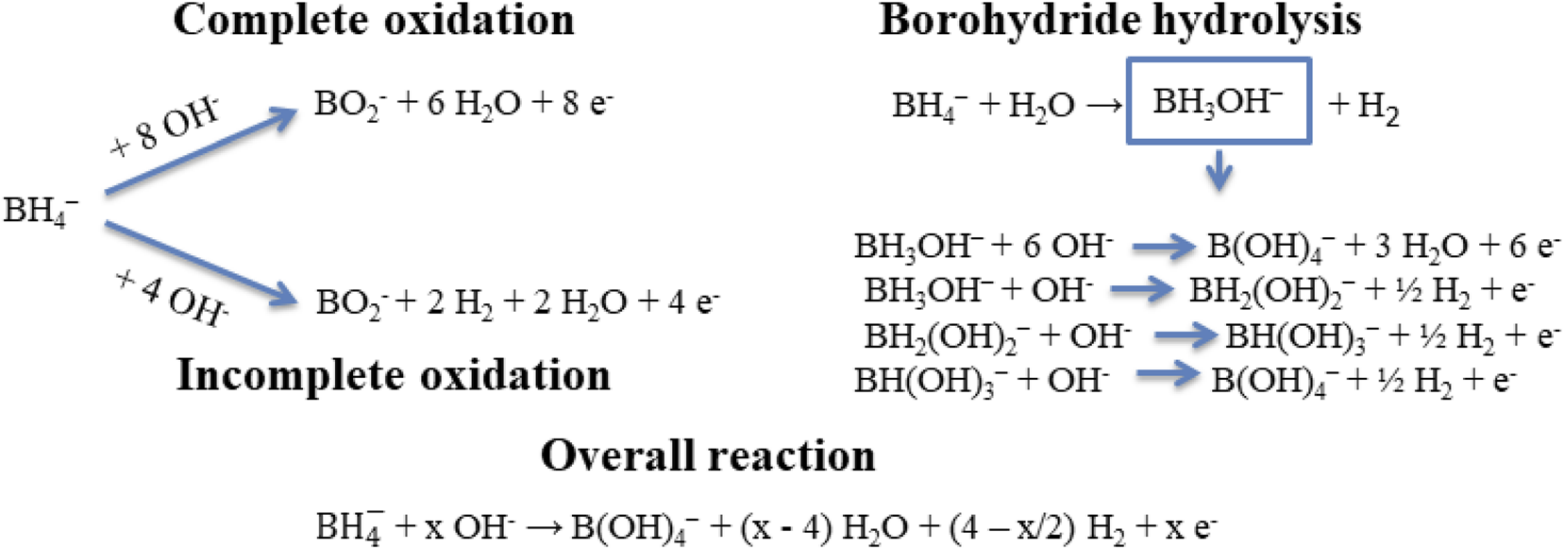
Schematic representation of the oxidation of BH_4_^−^ ions and their hydrolysis.

The electrochemical behavior of the Co and CoAu electrodes towards the oxidation of BH_4_^−^ ions was evaluated in an alkaline medium using cyclic voltammetry. Fig. 8a presents CVs of the Co and CoAu electrodes in a 1 M NaOH solution containing 0.05 M NaBH_4_ at 25 °C. Two anodic peaks: A0 (in the low-potential region from −1.2 to −0.6 V) and A (in the potential region from −0.5 to 0 V) were seen on the anodic scans for the Co and CoAu electrodes. The first peak (A0) was attributed to the oxidation of hydrogen, generated by the catalytic hydrolysis of BH_4_^−^ (eqn (2)) as well as to the oxidation of BH_4_^−^ ions (eqn (1)), while the second peak (A) was attributed to the direct oxidation of BH_4_^−^ ions. The mechanism of the mentioned reactions is rather complicated due to the formation of intermediates, adsorption phenomenon, and influence of the electrode potential, *e.g.*, formation, adsorption, and oxidation of H_2_ and BH_3_OH^−^, Scheme 3.^53–58^ It can be noted that during the cycling, current densities due to BH_4_^−^ ions oxidation are somewhat increased and stabilized. The current density of peak A (*ca.* −0.2 V) was *ca.* 7 times higher when using CoAu electrode than using Co electrode.

Fig. 8b presents positive-going potential scans of the CoAu electrode in the NaBH_4_ solution at 25 °C at different scan rates with a linear regression of peak current density *vs.* scan rate. The observed shift of peak potential to positive values with the increasing scan rate is typical for irreversible processes.^59^ Furthermore, BOR studies done in NaBH_4_ solutions of different concentrations (0.01–0.04 M range) revealed a linear increase of the current density of peak A with the increase of concentration along with a slight shift of peak potential. These results suggest that BOR proceeds as irreversible oxidation of a bulk species under mixed kinetic and diffusion control.

Fig. 8c and d shows the chronoamperometric curves recorded using Co and CoAu electrodes in NaBH_4_ solution at a fixed potential value of 0.85 V (Fig. 8c) and 0.05 V (Fig. 8d). At the end of the experimental period (*t* = 30 min), the current density values of the CoAu electrode are higher than those of Co electrode, indicating a higher electrocatalytic activity and stability of the prepared CoAu electrode towards the oxidation of BH_4_^−^. Namely, current densities at −0.85 V are about 1.4 and at 0.05 V are more than 300 times higher using CoAu electrode than using Co electrode (this ratio of current densities is in agreement with the voltammetry data). Furthermore, this initial 30 min *i*–*t* measurements demonstrated high stability of the prepared CoAu electrode. Thus comparison of the current density recorded at a potential of 0.05 V after 1 min and at the end of the experiment (after 30 min) revealed a decrease of only ∼8%. Comparison of current densities recorded at the same potential after 5 min and at the end of the experiment (after 30 min) showed a decrease as low as ∼1.3%.

**Fig. 9:**
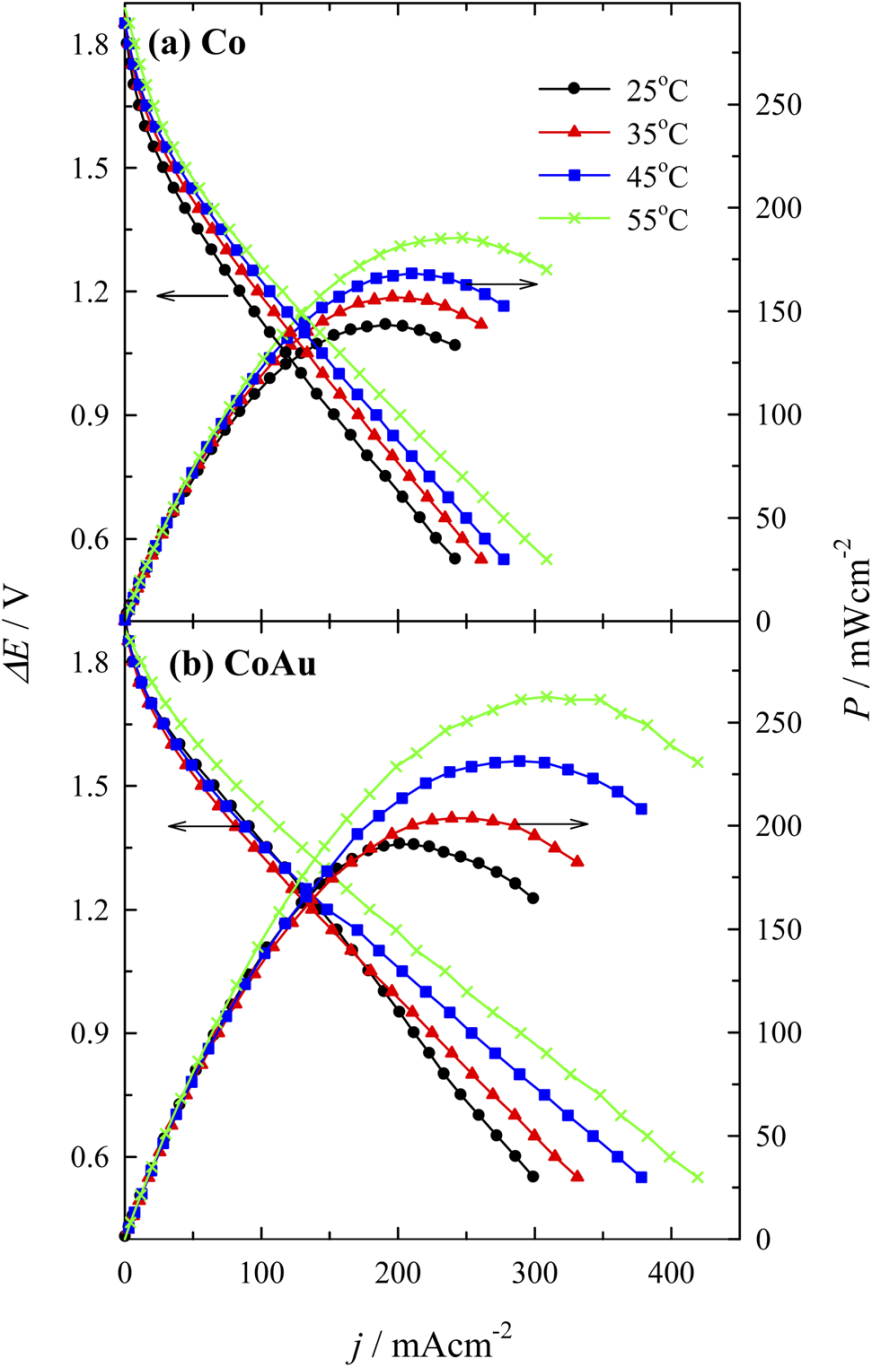
Cell polarization and power density curves for the DBHPFC using (a) Co and (b) CoAu anode at operating temperatures ranging from 25 to 55 °C.

Fuel cell measurements were performed using a homemade DBHPFC at 25, 35, 45, and 55 °C. During the cell discharge process, small bubbles of hydrogen and oxygen were observed at the electrodes' surface due to the chemical decomposition of BH_4_^−^ and H_2_O_2_ at the anode and at the cathode, respectively. Fig. 9 presents the fuel cell polarization curves and the corresponding power density curves *versus* the current density by employing the Co (Fig. 9a) and the CoAu (Fig. 9b) as an anode. The fuel cell displayed an open circuit voltage of *ca.* 1.9 V in both cases. However, it was found that power density is significantly higher in the case of the investigated CoAu anode when compared to that of Co anode (Fig. 9). Thus, peak power densities of 143 and 191 mW cm^−2^ were attained at 25 °C for DBHPFC with the Co and CoAu anode at a current density of 191 and 201 mA cm^−2^ and cell voltage of 0.75 and 0.95 V, respectively (Table 3). The peak power density increased 1.3 and 1.4 times with an increase in temperature from 25 °C to 55 °C using Co and CoAu anode, respectively.

**Table 3:** Comparison of the parameters of the DBHPFC employing Co and CoAu anodes with DBHPFCs employing Co- and Au-based anodes reported in the literature

Electrode	*T* (°C)	Anolyte	Catholyte	Cell voltage at peak power density (V)	Current density at a peak power density (mA cm^−2^)	Peak power density (mW cm^−2^)	Ref.
Co	25	1 M NaBH_4_ + 4 M NaOH	5 M H_2_O_2_ + 1.5 M HCl	0.75	191.19	143.47	This work
	35	1 M NaBH_4_ + 4 M NaOH	5 M H_2_O_2_ + 1.5 M HCl	0.80	195.88	156.78	This work
	45	1 M NaBH_4_ + 4 M NaOH	5 M H_2_O_2_ + 1.5 M HCl	0.80	210.10	168.19	This work
	55	1 M NaBH_4_ + 4 M NaOH	5 M H_2_O_2_ + 1.5 M HCl	0.75	247.12	185.46	This work
CoAu	25	1 M NaBH_4_ + 4 M NaOH	5 M H_2_O_2_ + 1.5 M HCl	0.95	201.19	191.25	This work
	35	1 M NaBH_4_ + 4 M NaOH	5 M H_2_O_2_ + 1.5 M HCl	0.80	254.31	203.56	This work
	45	1 M NaBH_4_ + 4 M NaOH	5 M H_2_O_2_ + 1.5 M HCl	0.80	288.99	231.34	This work
	55	1 M NaBH_4_ + 4 M NaOH	5 M H_2_O_2_ + 1.5 M HCl	0.85	308.68	262.60	This work
CoAu/rGO foam	30	2 M NaOH + 0.5 M NaBH_4_	2 M H_2_SO_4_ + 0.8 M H_2_O_2_	0.86	150	129	60
CoAu/Ni foam	30	2 M NaOH + 0.5 M NaBH_4_	2 M H_2_SO_4_ + 0.8 M H_2_O_2_	0.95	85	80.5	60
CoNi-NS/Ni foam	30	0.5 M NaBH_4_ + 4 M NaOH	0.8 M H_2_O_2_ + 2 M H_2_SO_4_	0.96	84	80.6	61
CoNi-NS/rGO foam	30	0.5 M NaBH_4_ + 4 M NaOH	0.8 M H_2_O_2_ + 2 M H_2_SO_4_	0.95	95.7	91.3	61
Co/C	25	1 M NaBH_4_ + 3 M NaOH	2 M H_2_O_2_ + 0.5 M H_2_SO_4_	1.05	45	47.5	28
Co/TiO_2_-NTs	25	1 M NaBH_4_ + 4 M NaOH	5 M H_2_O_2_ + 1.5 M HCl	0.80	108.7	87	62
Au–Y	25	1 M NaBH_4_ + 4 M NaOH	5 M H_2_O_2_ + 1.5 M HCl	0.5	295	150	28
	45	1 M NaBH_4_ + 4 M NaOH	5 M H_2_O_2_ + 1.5 M HCl	0.6	390	215	28
Au–Ni		0.5 M NaBH4 + 2 M NaOH	4.5 M H_2_O_2_ + 2 M HCl	0.57	130	74	63
Au_50_Fe_50_/C	25	1 M NaBH_4_ + 3 M NaOH	2 M H_2_O_2_ + 0.5 M H_2_SO_4_	0.50	69.6	34.9	64
Au_67_Fe_3_/C	25	1 M NaBH_4_ + 3 M NaOH	2 M H_2_O_2_ + 0.5 M H_2_SO_4_	0.51	64.8	32.9	64
Au/C	25	1 M NaBH_4_ + 3 M NaOH	2 M H_2_O_2_ + 0.5 M H_2_SO_4_	0.55	40.0	21.8	64
Au_49_Pd_51_/MWCNTs	35	5 wt% NaBH_4_, 10 wt% NaOH, and 85 wt% H_2_O	20 wt% H_2_O_2_, 5 wt% H_3_PO_4_, and 75 wt% H_2_O	—	—	191.1	65
	52	5 wt% NaBH_4_, 10 wt% NaOH, and 85 wt% H_2_O	20 wt% H_2_O_2_, 5 wt% H_3_PO_4_, and 75 wt% H_2_O	—	—	279.5	65
Au_74_Pd_26_/MWCNTs	35	5 wt% NaBH_4_, 10 wt% NaOH, and 85 wt% H_2_O	20 wt% H_2_O_2_, 5 wt% H_3_PO_4_, and 75 wt% H_2_O	—	—	149.1	65
Au/CNT-G	25	2 M NaBH_4_ + 6 M NaOH	2 M H_2_O_2_ + 1 M HCl	—	—	97	66
	40	2 M NaBH_4_ + 6 M NaOH	2 M H_2_O_2_ + 1 M HCl	—	—	106	66
	50	2 M NaBH_4_ + 6 M NaOH	2 M H_2_O_2_ + 1 M HCl	—	—	125	66
Au_45_Co_55_/C	25	1 M NaBH_4_ + 3 M NaOH	2 M H_2_O_2_ + 0.5 M H_2_SO_4_	0.78	85	66.50	29

Furthermore, the peak power density reached by the DBHPFC with CoAu anode is higher than that of DBHPFC with CoAu/rGO foam anode (129 mW cm^−2^ at 30 °C) and CoAu/Ni foam (80.5 mW cm^−2^ at 30 °C), Table 3.^60^ Similarly, a peak power density of DBHPFC with CoAu anode is higher than, for instance, that of a DBHPFC operating with Au–Y anode (150 mW cm^−2^ at 25 °C).^28^ Duan and co-workers reported that DBHPFC with Au–Ni anode catalyst showed a maximum power density of 74 mW cm^−2^ at 130 mA cm^−2^.^63^ Improved electrode kinetics for direct oxidation of BH_4_^−^ was reported for Au–Fe bimetallic catalysts (power density 34.9 mW cm^−2^) in comparison to the pure Au electrode (21.8 mW cm^−2^),^64^ but still lower than kinetics/power density reached herein.

## 4. Conclusions

Low Au-content CoAu 3D structured electrodes were prepared by simple electroless deposition and investigated for environmental applications. Thus, a detailed study on the electrochemical behaviour of CoAu electrode in the presence of As(iii) was performed. A peak corresponding to the oxidation of As(0) to As(iii) could be observed at *ca.* −0.36 V in a weakly alkaline medium, with a current density double that obtained using an Au electrode. The LOD of As(iii) sensing using CoAu electrode of 2.16 ppb was achieved under the optimized condition (deposition potential of −0.7 V, deposition time of 60 s) which is 5 times lower than the allowed WHO limit for arsenic in water. Furthermore, the presence of Cu(ii) as a model interferent did not affect the electrode's response to As(iii) presence. Finally, the analytical application of the prepared electrode toward the detection of As(iii) in real water samples was proven to be successful.

DBHPFC with CoAu anode attained a peak power density of 191 mW cm^−2^ at 25 °C at a cell voltage of 0.95 V, and it further increased 1.4 times with an increase in temperature to 55 °C.

Hence, the CoAu electrode could be used for various environmental applications, including the detection of trace amounts of As(iii) as well as BH_4_^−^ oxidation in DBHPFCs.

## Conflicts of interest

There are no conflicts to declare.

## Supplementary Material

RA-012-D2RA04828K-s001
