# Supplementary material for: Low Au-content CoAu electrodes for environmental applications
Source: RSC Adv. 2022 Sep 15;12(40):26134–46. doi: 10.1039/d2ra04828k (PMC9475402; doi:10.1039/d2ra04828k)
Supplement: RA-012-D2RA04828K-s001 [file RA-012-D2RA04828K-s001.pdf]

Electronic Supplementary Material (ESI) for RSC Advances.
This journal is © The Royal Society of Chemistry 2022

Low Au-content CoAu electrodes for environmental applications

Kristina Radinović[1], Jadranka Milikić[1], Aldona Balčiūnaitė[2], Zita Sukackienė[2], Marko Bošković,[3] Loreta Tamašauskaitė-Tamašiūnaitė[2] and Biljana Šljukić[1,4*]

[1]*University of Belgrade, Faculty of Physical Chemistry, Studentski trg 12-16, 11158 Belgrade, Serbia*

[2]*Center for Physical Sciences and Technology, Saulėtekio ave. 3, LT-10257 Vilnius, Lithuania*

[3] *University of Belgrade, Institute of Chemistry, Technology, and Metallurgy, Department of Microelectronic Technologies, Njegoševa 12, 11000 Belgrade, Serbia*

[4]*Center of Physics and Engineering of Advanced Materials, Laboratory for Physics of Materials and Emerging Technologies, Chemical Engineering Department, Instituto Superior Técnico, Universidade de Lisboa, 1049–001 Lisbon, Portugal*

* Corresponding author: biljka@ffh.bg.ac.rs

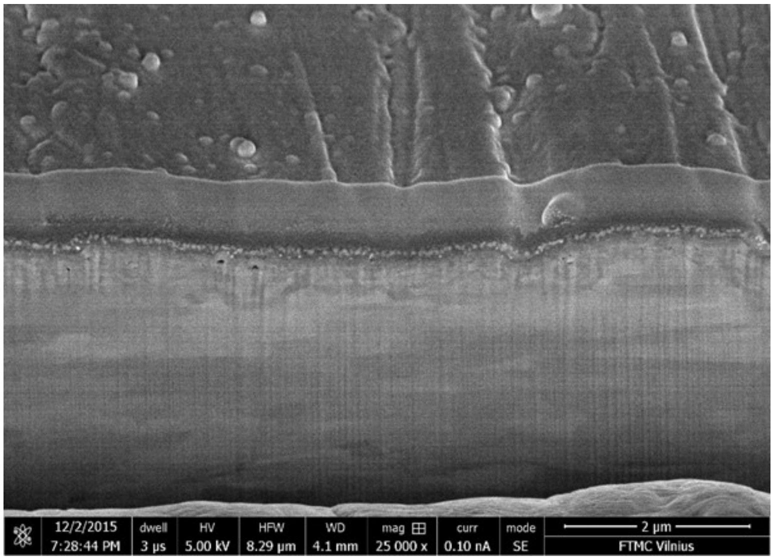

Figure S1. TEM image of the cross section of Co deposited onto Cu.

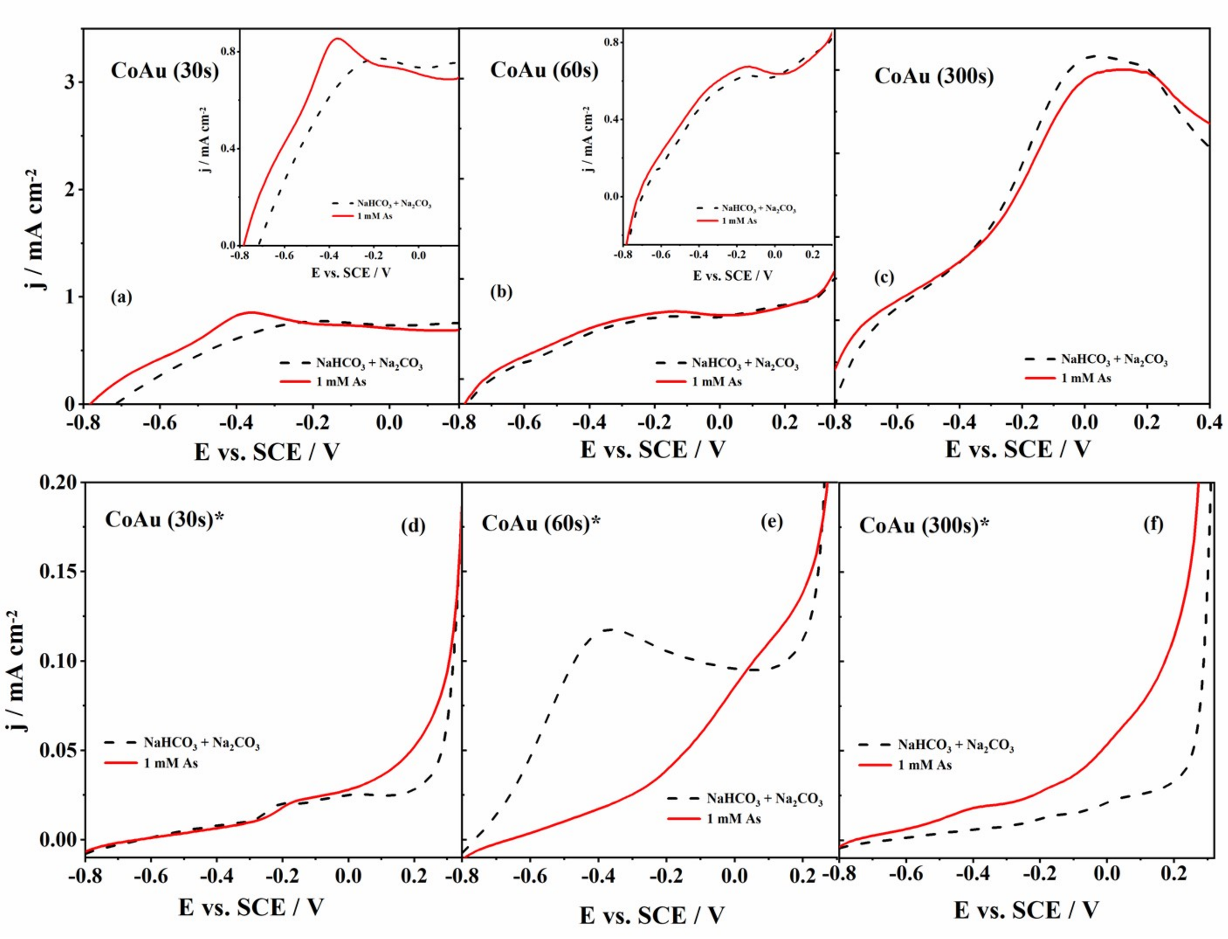

Figure S2. ASV of (a,b,c) CoAu electrodes prepared by immersion into 1 mM HAuCl4 (pH 1.8) at 30° C for different periods (30 s, 60 s and 300 s) and (d,e,f) CoAu electrodes prepared by immersion into 1 g 1^{-1} $KAu(CN)_2$ + 0.4 M $(NH_4)_2C_6H_6O_7$ complex (pH = 5) for various periods (30 s, 60 s and 300 s). ASVs recorded in $NaHCO_3$ + Na_2CO_3 buffer in the absence and in the presence of As (III) (1 mM) recorded at a scan rate of 50 mV s^{-1} after holding the potential at -0.7 V for 60 s.
